# Insonation versus Auscultation in Valvular Disorders: Is Aortic Stenosis the Exception? A Systematic Review

**DOI:** 10.5334/aogh.2489

**Published:** 2019-07-11

**Authors:** Dylan Stanger, Darryl Wan, Nima Moghaddam, Niki Elahi, Edgar Argulian, Jagat Narula, Amir Ahmadi

**Affiliations:** 1 Division of Cardiology, University of British Columbia, Vancouver, BC, CA; 2 Icahn School of Medicine at Mount Sinai Hospital, Mount Sinai Heart, New York, US

## Abstract

**Background:**

Handheld echocardiography is being proposed as the fifth pillar of bedside physical cardiovascular examination (PE) and is referred to as insonation. Although there is emerging consensus that insonation is superior to PE for diagnosis of various cardiac conditions, superiority has not been consistently demonstrated for various valvular heart disease (VHD) lesions. The objective of this review is to systematically review the accuracy of insonation and auscultation in published literature for detection of common VHD.

**Methods:**

An extensive literature search across three commonly used public databases allowed comparison of diagnostic characteristics of insonation and auscultation for common VHD including aortic stenosis, mitral regurgitation, aortic regurgitation, tricuspid regurgitation. Sensitivity, specificity, and accuracy of insonation and auscultation for the detection of these VHD lesions were extracted for further analysis. The quality of evidence was assessed according to Grading of Recommendations Assessment, Development and Evaluation (GRADE) methodology.

**Results:**

Eight hundred eighty studies were screened, and seven observational studies were selected for full analysis. Due to heterogeneity of data, this study was not amenable to meta-analysis. Insonation was superior to auscultation for the detection of all regurgitant lesions, but there was no significant difference in diagnostic ability of the two strategies for detection of aortic stenosis.

**Conclusions:**

Compared to auscultation, insonation, in its currently available form, is a superior diagnostic tool for regurgitant lesions. However, insonation fails to improve upon auscultation for recognition of aortic stenosis. This limitation is likely due to absence of spectral Doppler and inability of HE to assess transvalvular velocity and gradient.

## Introduction

Physical examination has remained the mainstay for bedside diagnosis of cardiovascular disease for centuries. Despite this, physical examination alone has limitations and has been subjected to increasing uncertainty in an era of evolving and advancing technology. In the last decade, handheld echocardiography (HE) has emerged as a novel and innovative adjunct to rapid diagnosis of disease [1,2,3].

The accuracy of auscultation for detection of cardiac pathology is variably subjective and highly performer dependent with low interobserver consistency [4,5]. Although physical examination is an indispensable component of patient bedside assessment, incorporation of new imaging technology such as portable cardiac ultrasound has allowed for enhanced point-of-care clinical diagnosis. The portability and low cost of HE has called for insonation to be a fifth pillar of the conventional examination, following inspection, palpation, percussion, and auscultation [6,7].

Recent American and European consensus guidelines have endorsed use of HE as a screening measure and an extension, not a replacement, to physical examination [8,9]. It has been demonstrated that HE findings correlate strongly with standard echocardiography [10]. HE offers significant diagnostic value in evaluation of inferior vena cava size, pericardial effusion, and morphological and functional assessment of cardiac chambers [11,12,13,14]. However, its role in diagnosing valvular heart disease (VHD) has not been well defined [15]. Prior studies have shown that HE can facilitate triage and early directed management of VHD [16,17,18]. Since HE is mainly limited to two-dimensional imaging and color Doppler, the accurate quantification of valve disease severity may be compromised. Furthermore, lack of spectral Doppler on HE can abate its accuracy in valvular stenosis.

In view of these facts, we conducted a systematic review of the available evidence on utility of HE in assessment of VHD. The purpose of this review was to: (1) synthesize the current literature examining both sensitivity and specificity of HE in VHD; (2) compare the diagnostic power of HE with auscultation; and (3) ascertain the limitations of HE in diagnosing VHD. We hypothesized that HE improves clinical assessment of valvular regurgitant lesions more robustly than stenotic lesions.

## Material and Methods

This systematic review was conducted as specified by Preferred Reporting Items for Systematic Reviews (PRISMA).

### Search strategies

We conducted a systematic search across multiple databases including MEDLINE Ovid, Cochrane Central Register of Controlled Trials, Cochrane Database of Systematic Reviews, and Embase to identify relevant articles published between January 1, 2000, to January 20, 2018. A medical librarian was consulted to optimize the search strategy and identify key search terms.

### Study selection

The abstracts of identified publications were screened by two independent reviewers. Potentially eligible articles were then selected for full-text review and compared between reviewers to ensure congruence. Original studies comparing point-of-care ultrasound, with or without physical examination, to traditional physical examination alone were included in this review. Animal studies, conference abstracts, and non-English studies were excluded.

### Data extraction

Selected studies were extracted in full text and relevant study data were recorded on an electronic data collection sheet. The primary objective was to obtain data regarding the accuracy of point-of-care ultrasound compared to physical examination alone in identifying valvular heart disease. Measured values for sensitivity and specificity of HE were extracted for stenotic and regurgitant lesions as compared to physical examination of the same lesions. In the majority of the selected studies, a valvular lesion was defined as clinically relevant when it was moderate or greater in severity.

### Quality assessment

Grading of Recommendations, Assessment, Development and Evaluation (GRADE) methodology was employed to determine the quality of evidence and the level of potential bias. In short, the GRADE approach assesses the combined quality of evidence for a given intervention by evaluating factors such as the risk of bias and any limitations of the data including incompleteness, imprecision, and indirectness. Using these parameters, the level of evidence for any given intervention was judged as being very low, low, moderate, or high. Two reviewers assigned level of bias and quality (DW and NM). Any discrepancies were resolved by a third-party reviewer (DS).

## Results

### Study and equipment characteristics

Overall, HE was performed on 2090 patients across all the reviewed studies. The level of training varied in these studies from medical students, to hospitalists, to cardiologists. For the non-cardiologists, didactic and hands-on training (i.e. ultrasound principles, cardiac anatomy and function, image acquisition) was completed to ensure overall competence in performing HE. Although different pocket-sized technologies were selected in these studies, all handheld devices used were two-dimensional echocardiographic applications with full-scale color flow. However, none these devices had spectral Doppler capabilities. The details of the reviewed studies are outlined in Table 1. In the majority of the studies, valvular pathologies identified through standard echocardiography were used as the gold standard.

**Table 1:** Summary of Handheld Echocardiography Operator and Equipment Characteristics.

Study	Lesion	N	Provider level	Level of training	Equipment	US Capability

Godown et al. 2015	AR MR	1317	Cardiologist	Cardiologist	Vscan (GE)	2D, colour Doppler
Kobal et al. 2005	AS AR MR TR	61	Medical student	4 hours lecture 14 hours hands-on	Philips OptiGo	2D, colour Doppler
Martin et al. 2009	AS AR MR	354	Hospitalist	Performed 5 echocardiograms, 6 hours spent on interpretation	SonoSite Elite	2D, colour Doppler
Mehta et al. 2014	AS MR TR	250	Cardiologist	Cardiologist (<2 to >20 years)	Vscan (GE Healthcare)	2D, colour Doppler
Spencer et al. 2001	AS AR MR TR	36	Cardiologist	Cardiologist, level 2 (average 5 years’ practice)	Agilent Technologies	2D, colour Doppler
Stokke et al. 2014	AS AR MR	72	Medical student	4 hours training program	Vscan (GE Vingmed)	2D, colour Doppler

N: number of patients examined; US: Ultrasounds.

### Aortic stenosis

Four prospective cohort studies compared the efficacy of HE with physical exam in diagnosing aortic stenosis (AS) [2,19,20,21,22]. The sensitivity and specificity of HE in identifying AS ranged from 62%–94% and 85%–98%, respectively. These ranges were comparable to auscultation (Table 2). Although there was substantial inter-rater agreement in evaluation of AS in most studies, hand-carried ultrasound failed to improve the diagnostic accuracy of AS. In one study, HE performed by internists underestimated findings of AS compared to traditional echocardiography read by cardiologists [19]. Even amongst expert cardiologists, HE was equivalent to physical examination in excluding or diagnosing AS [2].

**Table 2:** Summary of reviewed studies in aortic stenosis.

Study	Handheld Echocardiography			Physical Examination			p-value

	Sensitivity	Specificity	Accuracy/concordance	Sensitivity	Specificity	Accuracy/concordance	

Kobal et al. 2005	**0.62**	NR	NR	**0.46**	NR	NR	Sens: NS
Martin et al. 2009	**0.79** [0.75–0.84]	**0.85** [0.81–0.89]	**0.82** [0.76–0.87]	**0.68** [0.55–0.81]	**0.94** [0.91–0.97]	**0.81** [0.75–0.86]	Spec: NS
Mehta et al. 2014	**0.94** [0.67–0.99]	**0.98** [0.95–0.99]	NR	**0.88** [0.61–0.97]	**0.97** [0.93–0.98]	NR	Sens: 1.0 Spec: 0.55
Stokke et al. 2014	**0.70** [0.5–0.86]	**0.97** [0.87–0.99]	**0.71**	**0.67** [0.46–0.83]	**0.98** [0.9–1.0]	**0.71**	Sens: NS

[ ]: confidence intervals; NR: not reported; NS: not significant; Sens: sensitivity; Spec: specificity.

Overall, low-quality evidence demonstrates no statistically significant advantage with HE in comparison to physical examination in detection of AS (Table 2).

### Aortic regurgitation

Physical examination has limited ability in assessment of diastolic murmurs, with sensitivities as low as 14%–33% for diagnosing aortic regurgitation (AR) [21,22,23]. In all five prospective cohort studies comparing HE to physical exam in evaluation for AR, HE consistently improved detection of AR with slight overestimation in its diagnosis and lesion severity (see Table 3). The overall accuracy and concordance of HE for recognition of AR remained robust (κ = 0.75–0.85). However, in one study, HE was less accurate in detecting absence of AR compared to physical examination (specificity 59% vs. 86%, p < 0.001) [19].

**Table 3:** Summary of reviewed studies in aortic regurgitation.

Study	Handheld Echocardiography			Physical Examination			p-value

	Sensitivity	Specificity	Accuracy/concordance	Sensitivity	Specificity	Accuracy/concordance	

Godown et al. 2015	**0.818**	**0.992**	NR	**0.136**	**0.998**	NR	Sens: <0.001
Kobal et al. 2005	**0.92**	**0.78**	**0.85**	**0.49**	**0.81**	**0.67**	Sens: <0.001
Martin et al. 2009	**0.76** [0.67–0.85]	**0.73** [0.67–0.79]	**0.745** [0.68–0.80]	**0.69** [0.59–0.79]	**0.89** [0.85–0.94]	**0.79** [0.673–0.84]	Spec 0.001 Sens: NS
Spencer et al. 2001	**0.78**	NR	NR	**0.26**	NR	NR	Sens: <0.05
Stokke et al. 2014	**0.43** [0.23–0.66]	**0.90** [0.80–0.96]	κ = **0.36**	**0.33** [0.15–0.57]	**1.0** [0.94–1.0]	κ = **0.44**	Sens: NS

[ ]: confidence intervals; NR: not reported; NS: not significant; Sens: sensitivity; Spec: specificity.

Overall, very low-quality evidence supports superiority of HE to physical examination in identification of AR (Table 3).

### Mitral regurgitation

Six prospective cohort studies evaluated the role of HE in detecting mitral regurgitation (MR) (see Table 4). With the exception of one study [22], HE was markedly superior in screening for MR with a significantly higher sensitivity compared to physical examination. Spencer et al. demonstrated that auscultation failed to correctly identify 23% of patients with MR with two-fold improvement in diagnosis with utilization of HE [8]. However, the overall specificity of physical exam is comparable to HE. Martin et al. showed that although HE has better accuracy than clinical exam in detection of MR (50% vs. 35%, p = 0.001), it is less accurate in detecting its absence (54% vs. 79%, p = 0.0001) [19]. Additionally, the concordance of mitral regurgitation detection was lower in trainees than in expert operators with a kappa statistics for agreement (κ) of 0.59 and 0.82, respectively [2,20].

**Table 4:** Summary of reviewed studies in mitral regurgitation.

Study	Handheld Echocardiography			Physical Examination			p-value

	Sensitivity	Specificity	Accuracy/concordance	Sensitivity	Specificity	Accuracy/concordance	

Godown et al. 2015	**0.532**	**0.966**	NR	**0.159**	**0.915**	NR	Sens: 0.31
Kobal et al. 2005	**0.92** [0.851–0.962]	**0.78** [0.70–0.85]	**0.85** [0.79–0.89]	**0.49** [0.39–0.59]	**0.81** [0.73–0.87]	**0.67** [0.60–0.73]	Sens: <0.001 Spec: NS
Martin et al. 2009	**0.76** [0.69–0.82]	**0.80** [0.72–0.87]	**0.78** [0.72–0.84]	**0.70** [0.63–0.77]	**0.89** [0.83–0.94]	**0.795** [0.73–0.85]	Spec: 0.05 Sens: NS
Mehta et al. 2014	**1.0** [0.97–1.0]	**0.996** [0.97–1.0]	NR	**0.6** [0.54–0.66]	**0.97** [0.94–0.99]	NR	Sens: 0.008 Spec: 0.07
Spencer et al. 2001	**0.785**	NR	NR	**0.532**	NR	NR	Sens: <0.05
Stokke et al. 2014	**0.69** [0.53–0.82]	**0.89** [0.78–0.95]	**0.59**	**0.29** [0.16–0.45]	**0.90** [0.76–0.96]	**0.21**	Sens: <0.01

[ ]: confidence intervals; NR: not reported; NS: not significant; Sens: sensitivity; Spec: specificity.

Overall, low-quality evidence supports the incorporation of HE in clinical assessment of patients with MR (Table 4).

### Tricuspid regurgitation

Three prospective cohort studies directly compared HE to physical examination for evaluation of tricuspid regurgitation (TR) [2,19,21]. Similar to other regurgitant lesions, HE had improved sensitivity in diagnosis TR with a value of 69%–92% compared to 28%–49% for physical examination. In one study, HE resulted in approximately three-fold increase in correct diagnosis of TR [19]. Mehta et al. demonstrated similar efficacy for HE in excluding TR relative to physical exam but clear superiority in diagnosing moderate to severe TR [2].

Overall, low-quality evidence illustrates that HE is a valuable tool in detection of TR (Table 5).

**Table 5:** Summary of reviewed studies in tricuspid regurgitation.

Study	Handheld Echocardiography			Physical Examination			p-value

	Sensitivity	Specificity	Accuracy/concordance	Sensitivity	Specificity	Accuracy/concordance	

Kobal et al. 2005	**0.92** [0.85–0.96]	**0.78** [0.70–0.85]	**0.85** [0.79–0.89]	**0.49** [0.39–0.59]	**0.81** [0.73–0.87]	**0.67** [0.60–0.73]	Sens: <0.001 Spec: NS
Mehta et al. 2014	**0.88** [0.69–0.96]	**0.97** [0.94–0.99]	NR	**0.28** [0.14–0.48]	**0.98** [0.95–0.99]	NR	Sens: <0.001 Spec: 0.75
Spencer et al. 2001	**0.691**	NR	NR	**0.345**	NR	NR	<0.05

[ ]: confidence intervals; NR: Not reported; NS: Not significant; Sens: sensitivity; Spec: specificity.

## Discussion

The purpose of this review was to rigorously evaluate and summarize, using GRADE methodology, the available evidence underpinning the utility of HE in assessment of VHD, focusing on the comparison of diagnostic power of HE compared to physical examination.

Consensus guidelines endorse use of HE as a screening measure and an extension, not a replacement, to physical examination [8]. Recently, insonation has been proposed as the fifth pillar of bedside physical cardiovascular examination. Although there is emerging consensus that insonation is superior to PE for diagnosis of various cardiac conditions, superiority has not been clearly demonstrated for various valvular heart disease (VHD) lesions.

Our review examined the diagnostic power of HE compared to physical examination with respect to four common valvular lesions – aortic stenosis, aortic regurgitation, mitral regurgitation, and tricuspid regurgitation. We demonstrated that there is low-quality evidence showing no statistically significant advantage of HE in comparison to physical examination in detection of AS. In the four included studies, there was considerable heterogeneity in performer skillset in HE (i.e. from medical student, to internist, to cardiologist) and a lack of statistical power due to small sample. Ultimately, these limitations preclude important comparisons. Evidence was also insufficient to draw definitive conclusions from prospective studies with absence of randomization. Furthermore, none of the studies adjusted for potential confounders.

Very low-quality evidence supports superiority of HE to physical examination in identification of AR. In general, physical examination has limited ability in assessment of diastolic murmurs. Cohort studies comparing HE to physical exam in evaluation for AR demonstrated that HE consistently improved detection of AR. The overall accuracy and concordance of HE for recognition of AR remained robust. The inconsistencies in quantifying severity of AR were partially attributed to inadequate training of HE performers with respect to the acquisition of color-flow Doppler images [19,20]. Despite the low strength of evidence, current literature supports the diagnostic utility of HE for AR in combination with clinical exam when performed by individuals with prior training in echocardiography.

With respect to evaluation of MR, low-quality evidence suggests that HE is markedly superior in screening for MR with a significantly higher sensitivity compared to physical examination. The enhanced diagnostic ability of HE is further pronounced in patients with severe left ventricular dysfunction, a clinical scenario where physical exam is known to more frequently miss or underestimate MR severity [20]. Further, specificity of physical exam is comparable to HE. As anticipated, the concordance of mitral regurgitation detection was lower in trainees than in expert operators. This is a somewhat expected finding as both qualitative and quantitative methods of evaluating the severity of MR, specifically with HE but also formal echocardiography, may be compromised by the presence of a noncircular cross-sectional jet profile, eccentric direction of flow into the left atrium with marked Coanda effect, dynamic regurgitant orifice, and multiple jets [2,20]. However, even in individuals with various levels of training and experience, HE greatly improved their diagnostic performance for MR beyond physical examination.

Overall, low-quality evidence illustrates that HE is a valuable tool in detection of TR. As with MR, HE had improved sensitivity in the diagnosis of TR when compared to physical examination. However, definitive conclusions cannot be attained due to small sample size of patients with TR in these studies (cumulative n = 105).

Our review has several limitations. First, all of the included studies have inherent significant inter-study heterogeneity, and all of the data comes from observational studies rather than RCTs. Second, for practical reasons, we restricted our search to English-language studies, and thus, may have omitted some relevant studies. Third, as with all systematic reviews, there is potential for publication bias as studies with positive findings are more likely to be published than those with negative findings. Lastly, we were unable to confirm the consistency of valvular diagnoses across the studies. Although the cut-off for clinically relevant lesions were predominantly established as moderate or greater, a few studies included mild valvular pathology in the total number of detected lesions. This could potentially affect the calculated sensitivity and specificity.

In conclusion, insonation in its currently available form is more diagnostically useful in the identification of regurgitant lesions. However, insonation fails to improve upon auscultation for recognition of aortic stenosis. This limitation is likely due to absence of spectral Doppler and inability of HE to assess transvalvular velocity and gradient.
